# Supplementary material for: Biochemical and Functional Profiling of Thioredoxin-Dependent Cytosolic GPX-like Proteins in Euglena gracilis
Source: Biomolecules. 2024 Jun 27;14(7):765. doi: 10.3390/biom14070765 (PMC11275057; doi:10.3390/biom14070765)
Supplement: Supplementary file 1 [file biomolecules-14-00765-s001.zip › biomolecules-3037571-supplementary.docx]

**
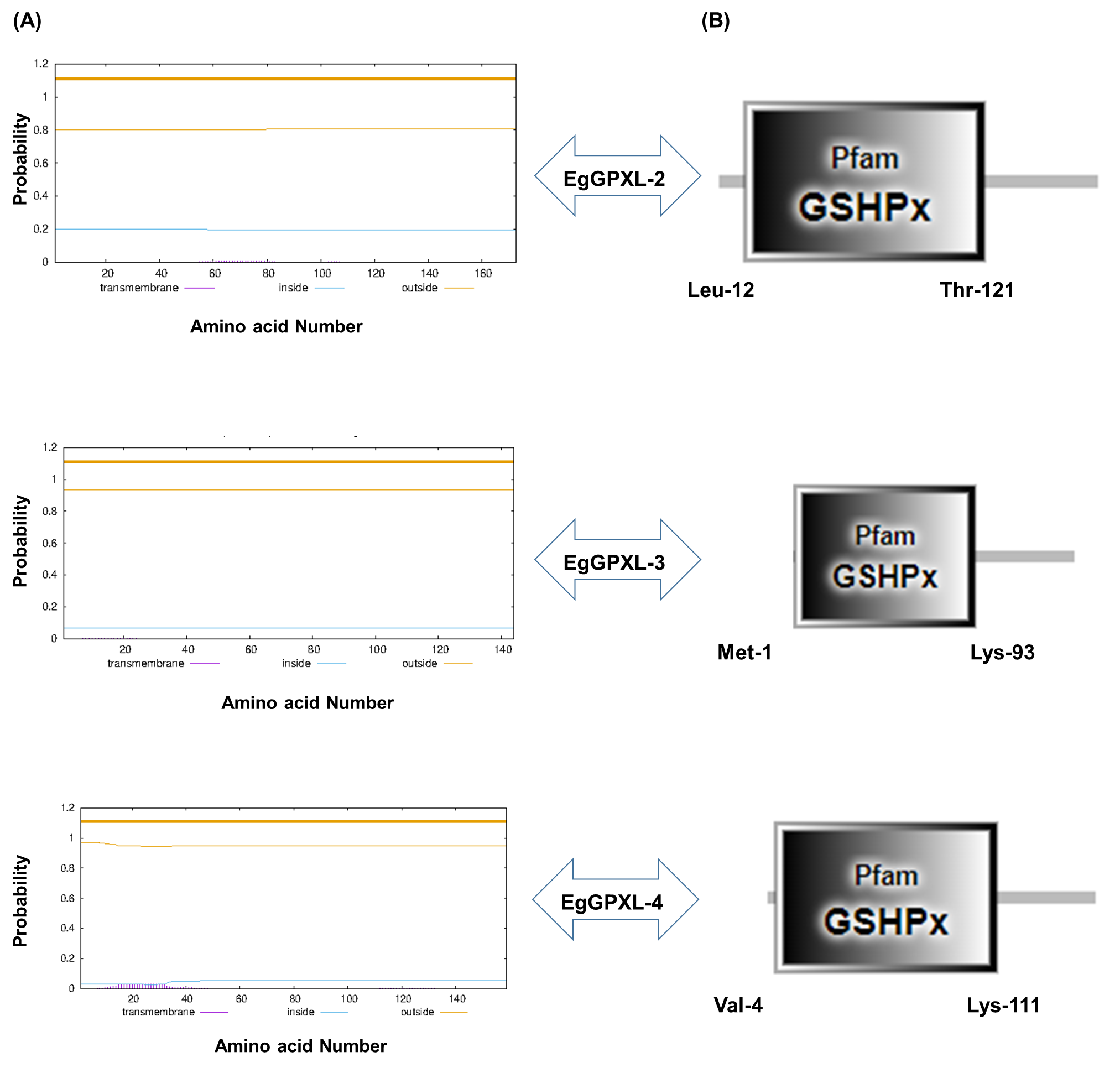
**

**Supplementary Fig 1:** Prediction of domains region in EgGPXLs. (A) No transmembrane region was detected in any EgGPXLs (using TMHMM program (<https://services.healthtech.dtu.dk/services/TMHMM-2.0/>)), indicating the possibility of cytosolic protein. (B) In addition, the characteristic GSHPx (Glutathione peroxidase domain) was found in all the EggPXLs Domain analysis conducted using SMART (http://smart.embl-heidelberg.de/), accordance with the previous studies [26, 27].

27].SMART (http://smart.embl-heidelberg.de/), accordance with the previous studies [26, 27].**
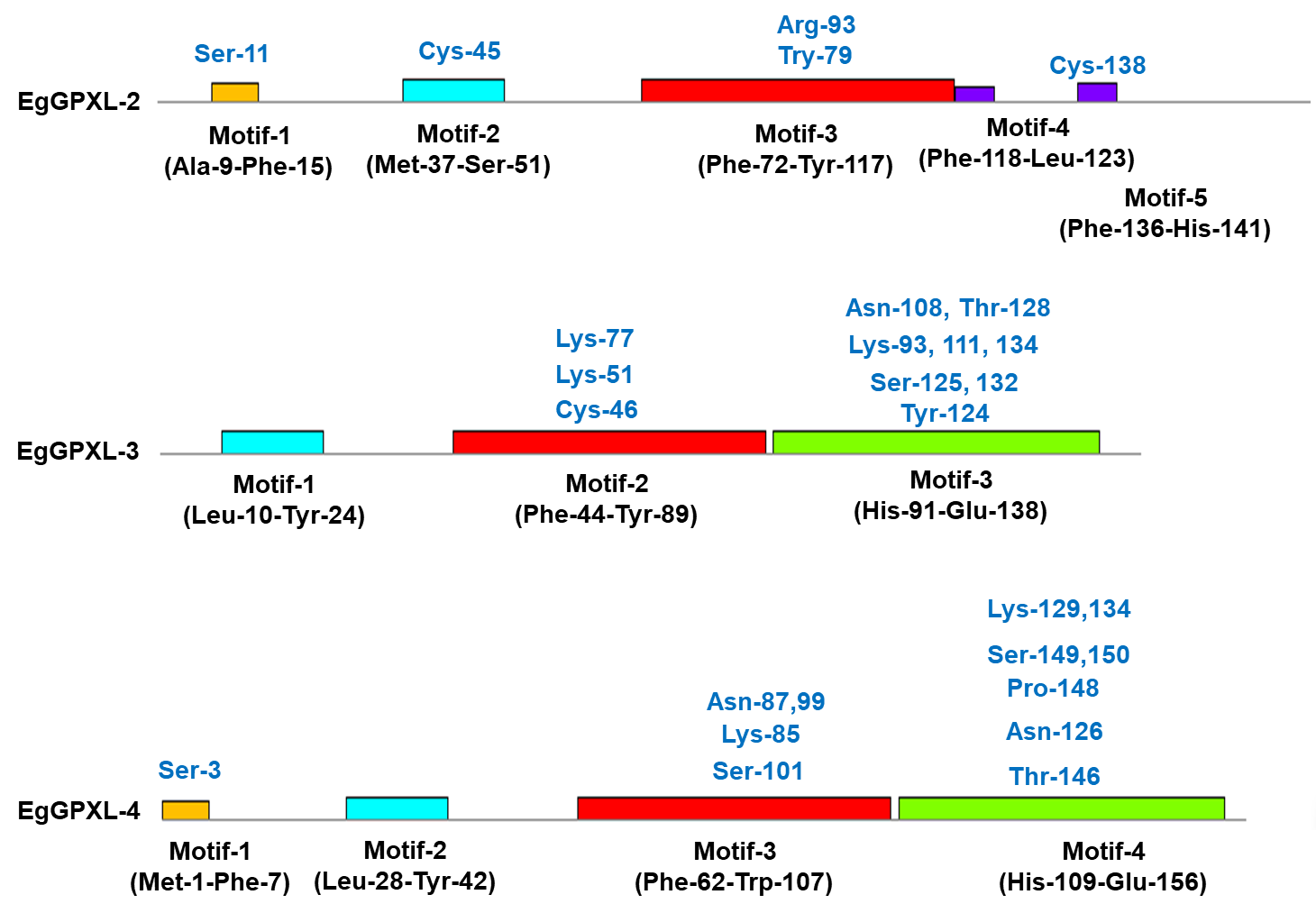
**

**Supplementary Fig 2:** Motif analysis of EgGPXLs. Motif analysis was conducted using MEME tool (Version 4.11.2) (<http://meme-suite.org/tools/meme>). MEME analysis anticipated five, three, and four motif in EgGPXL-2, EgGPXL-4, and EgGPXL-3, respectively. Different color (orange, cyan, red, purple, and green) indicated the different type of motif obtained from these cytosolic EgGPXLs based on sequence analysis. Further, some PTM sites (Illustrated as blue color) were also predicted in these motifs excepting motif-4, motif-1, motif-2 of EgGPXL-2, EgGPXL-4, and EgGPXL-3, respectively.

| **Supplementary Table S1:** Primers used in this study | |
| --- | --- |
| **Name** | **Sequence** |
| EgGPXL-2FP-Infusion | 5′-GAG CTC GGTACCCTCGGC TTGCCCTCCATGAGTAAC-3′ |
| EgGPXL-2RP-Infusion | 5′-GACAAGCTTGAATTCCTTACAAACGTGAGCTGGGGT TC-3′ |
| EgGPXL-3FP-Infusion | 5′-GAGCTCGGTACCCTCATGGCGGAGTTCAAGGGCAAGGT-3′ |
| EgGPXL-3RP-Infusion | 5′-GACAAGCTTGAATTCACAGCCCACACCCTAAGCACACC-3′ |
| EgGPXL-4FP-Infusion | 5′- GAGCTCGGTACCCTCCCATCTGTCCACGATTTCTCAG-3′ |
| EgGPXL-4RP-Infusion | 5′- GACAAGCTTGAATTCCTCACAGCAGCTTCTCGATGTCC-3′ |
| Prx1-T7-FP-RNAi | 5′- TAATACGACTCACTATAGGGTCAACG GGGAGTTCAAG-3′ |
| Prx1-T7-RP-RNAi | 5′- TAATACGACTCACTATA GGGGGGATCTGCCTTCATCG-3′ |
| Prx4-T7-FP-RNAi | 5′- TAATACGACTCACTATAGGGTACCGCTGGCAG GAC-3′ |
| Prx4-T7-RP-RNAi | 5′- TAATACGACTCACTA TAGGGGGCGTCCGACACCTC-3′ |
| GPXL2-T7-FP-RNAi | 5′- TAATACGACTCACTATAGGGGGAAAGCTGACAATGGTGGT-3′ |
| GPXL2-T7-RP-RNAi | 5′- TAATACGACTCACTATAGGGAATCTCGTCCCAGTTGTTGG-3′ |
| GPXL3-T7-FP-RNAi | 5′-TAATACGACTCACTATAGGGAGCATCAAGGCGGACATCGAGC-3′ |
| GPXL3-T7-RP-RNAi | 5′-TAATACGACTCACTATAGGGAGAGGGGAGGAACGAGGGCACTAA-3′ |
| GPXL4-T7-FP-RNAi | 5′-TAATACGACTCACTATAGGGCCACGATTTCTCAGTGCTCA-3′ |
| GPXL4-T7-RP-RNAi | 5′-TAATACGACTCACTATAGGGGTCTTCAGGTGCTCCCACAG-3′ |
| EF1α-FP | 5′-ACAGATTGGGAACGGGTACGC-3′ |
| EF1α-RP | 5′-CGCAGTTTCCCTTCACCATCG-3′ |

| **Supplementary Table S2:** Electroporation condition used in this study | | | | | | |
| --- | --- | --- | --- | --- | --- | --- |
|  | **Voltage (V)** | **Pulse length (msec)** | **Pulse interval (msec)** | **Number of pulse** | **Decay rate (%)** | **Polarity** |
| Poring pulse | 250 | 3 | 50 | 2 | 10 | + |
| Transfer pulse | 20 | 50 | 50 | 5 | 40 | +/- |

| **Supplementary Table S3:** Predicted post-translational modification (PTM) sites in cytosolic EgGPXLs. PTM sites were anticipated using MusiteDeep PTM prediction server (https://www.musite.net/). | | | |
| --- | --- | --- | --- |
| **Post translational modification** | **Amino acid residues/sequence** | | |
|  | EgGPXL-2 | EgGPXL-3 | EgGPXL-4 |
| Phosphorylation | S2, S11,Y164 | S125,S132 | S3,S8,S101,T146,S149,S150 |
| Glycosylation |  | N108,T128 | N87,N99,N126,T146 |
| Methylation | R65,R93, | K5,K134 | - |
| Palmitoylation | - | C46 | - |
| SUMOylation | - | K51,K93,K134 |  |
| Acetylation | K28 | K77,K111 | K85,K129,K134 |
| Hydroxylation | - | - | P148 |
| Ubiquitination | - |  | K134 |
| S-nitrosylation | Cys-45, 138 | Cys-100 | - |
| Nitration | Tyr-52,59,62  Try-79 | Tyr-34, 124 | - |
